# Delirium Tremens

**Published:** 1845-11

**Authors:** 


*Delirium Tremens.*—The following remarkable case of delirium tremens, is given by Mr. S. Flood, in which, after trying opium fully, with tartar emetic, digitalis, &c., without effect, belladonna was employed in the following way. A large plaster having been applied between the scapulæ, the cuticle was stripped off, three inches long and two wide, and a plaster of pure extract of belladonna applied to the denuded surface. The man was, at the time, in a state of furious delirium, with contracted pupils, pulse 100, weak, and very irritable; and had not slept for 360 hours. So acute was the pain produced by the plaster, that he was instantly subdued; and entreated its immediate removal. In three minutes he ceased to complain; in five minutes there were slight twitchings of the muscles of the face and arms, his utterance became indistinct, and he kept up a stupified laugh like a man much intoxicated; the pupils began rapidly to dilate, and in seven minutes were open to their fullest extent. He now became very drowsy and begged to lie down; the belladonna, therefore, was sponged off, simple ointment applied, and he then fell back on his pillow, and in nine minutes from the first application was in a profound sleep, which lasted for seven hours. During the sleep, which was free from stertor, the pulse fluctuated remarkably. At the commencement it was 110, small and irritable; in five minutes it rose to 140; and in twenty minutes to 160; then gradually fell, till at the end of six hours, it had sunk to 108, and was full and soft. At the end of seven hours he awoke quite quiet, but after staring about him in stupified astonishment, soon relapsed into his former state of wildness. Opiates were now tried again in large doses, but without effect, and as he was apparently sinking from prolonged excitement, belladonna was applied, in the same way, a second time, two days after the first application. The same chain of phenomena followed, and sound sleep was induced, which continued for nine hours and a half. On the following day, belladonna was a third time applied, but to the same surface; and from this time he gradually improved. Dr. Fosgate* recommends the union of ammonia with opium, not only as aiding to sustain the powers of the system, but also as modifying the influence of opium, diminishing its poisonous, and increasing its theraputic action.—*B. & F. M. Rev.*

